# Perturbing low dimensional activity manifolds in spiking neuronal networks

**DOI:** 10.1371/journal.pcbi.1007074

**Published:** 2019-05-31

**Authors:** Emil Wärnberg, Arvind Kumar

**Affiliations:** 1 Dept. of Computational Science and Technology, School of Electrical Engineering and Computer Science, KTH Royal Institute of Technology, Stockholm, Sweden; 2 Dept. of Neuroscience, Karolinska Institutet, Stockholm, Sweden; UCL, UNITED KINGDOM

## Abstract

Several recent studies have shown that neural activity *in vivo* tends to be constrained to a low-dimensional manifold. Such activity does not arise in simulated neural networks with homogeneous connectivity and it has been suggested that it is indicative of some other connectivity pattern in neuronal networks. In particular, this connectivity pattern appears to be constraining learning so that only neural activity patterns falling within the intrinsic manifold can be learned and elicited. Here, we use three different models of spiking neural networks (echo-state networks, the Neural Engineering Framework and Efficient Coding) to demonstrate how the intrinsic manifold can be made a direct consequence of the circuit connectivity. Using this relationship between the circuit connectivity and the intrinsic manifold, we show that learning of patterns outside the intrinsic manifold corresponds to much larger changes in synaptic weights than learning of patterns within the intrinsic manifold. Assuming larger changes to synaptic weights requires extensive learning, this observation provides an explanation of why learning is easier when it does not require the neural activity to leave its intrinsic manifold.

## Author summary

A network in the brain consists of thousands of neurons. A priori, we expect that the network will have as many degrees of freedom as its number of neurons. Surprisingly, experimental evidence suggests that local brain activity is confined to a subspace spanned by ~10 variables. Here, we employ three established approaches to construct spiking neuronal networks that exhibit low-dimensional activity. Using these models we address a specific experimental observation, namely that monkeys easily can elicit any activity within the subspace but have trouble with any activity outside. Specifically, we show that tasks that requires animals to change the network activity outside the subspace would entail large changes in the neuronal connectivity, and therefore, animals are either slow or not able to acquire such tasks.

## Introduction

The availability of novel experimental methods allows for simultaneous recording of 1000s of neurons and has made it possible to observe the fine structure of temporal evolution of task-related neuronal activity *in vivo*. The multi-unit neuronal activity can be described in terms of an *N* dimensional neural state-space where each axis (typically) corresponds to the firing rate of each neuron. The activity at a particular time corresponds to a point in this space, and the temporal evolution of the neuronal activity constitutes a trajectory. Analysis of such trajectories has revealed that across different brain regions and in different behavioral conditions the neural activity remains low dimensional [1–7] (but see [8]) such that the dimensionality of the activity is much smaller than number of neurons. That is, the trajectories corresponding to the task-related activity tend to be constrained to a linear subspace (the *intrinsic manifold*) of the state space rather than moving freely in all directions.

The empirical importance of the relation between dimensionality of activity and network connectivity was best illustrated by an experiment involving brain-computer-interface (BCI) learning in monkeys. Sadtler et al. [4] showed that animals were able to quickly learn the BCI task when the neural activity mapping was confined to the intrinsic manifold. By contrast, BCI learning was much slower (or non-existent) when the neural activity mapping for the BCI task was outside the intrinsic manifold [4]. Here, we address the question of why relearning activity patterns is easier within the intrinsic manifold than outside. Note that our goal is to build a conceptual understanding and not to faithfully model every aspect of the experiment.

Given that the neural activity appears to be low dimensional, Gallego et al. [9] recently proposed a division of the neural dynamics into a set of *latent variables* and a set of *neural modes*. The neural modes are static over the relevant timescales and are identified as factor loadings or principal components of the neural activity. Each neural mode is associated with a latent variable so that the actual neural activity at any point in time is a sum of the neural modes weighted by the respective latent variable. This description is analogous to the intrinsic manifold described in [4]: the neural modes correspond to the vectors spanning the intrinsic manifold.

To analyze perturbations of the intrinsic manifold, we first need a neural network that exhibits low-dimensional activity. A trivial solution would be to drive a recurrent network with low-dimensional input. However, this solution does not address the problem of manifold generation and merely assumes that an unspecified upstream network can generate a low-dimensional input. Therefore, here we make the assumption that low dimensionality is enforced by the local circuitry. In particular, we assume that the specific neural modes arise from local circuitry and that structured inputs may influence the values and trajectories of the latent variables but not the neural modes themselves. Following these assumptions, we ask what changes of the local network are required to change the neural modes, i.e. to rotate the intrinsic manifold. In particular, what type of changes in the neural modes requires the largest changes of local synaptic weights? Under the assumption that changing synaptic weights requires application of some learning rule, this may serve as an unbiased proxy for learning difficulty.

A network that exhibits a *D*-dimensional line attractor dynamics is one of the simplest network models that fulfills the above criteria and maintains *D* neural modes. By definition a line attractor will ensure that the activity remains in the *D*-dimensional intrinsic manifold. There are several frameworks for creating line attractors and networks with dynamical latent variables [10]. Here, we focus on three popular frameworks: (1) FORCE-learning [11, 12], (2) The Neural Engineering Framework (NEF; [13, 14]) and (3) Efficient Coding networks [15, 16]. We show that for all three frameworks, neural modes directly correspond to the *encoders* which are specified when the system is defined. Thus, we identified a feature common to all three frameworks. Importantly, we show that irrespective of the choice of modeling framework, rotating the neural modes within the intrinsic manifold only requires minor changes in the local synaptic weights, while rotating the neural modes outside the intrinsic manifold requires a complete rewiring of the network. This provides a possible explanation for Sadtler et al.’s observation [4] and offers new insights into the functional role of neural modes.

## Results

**Fig 1:**
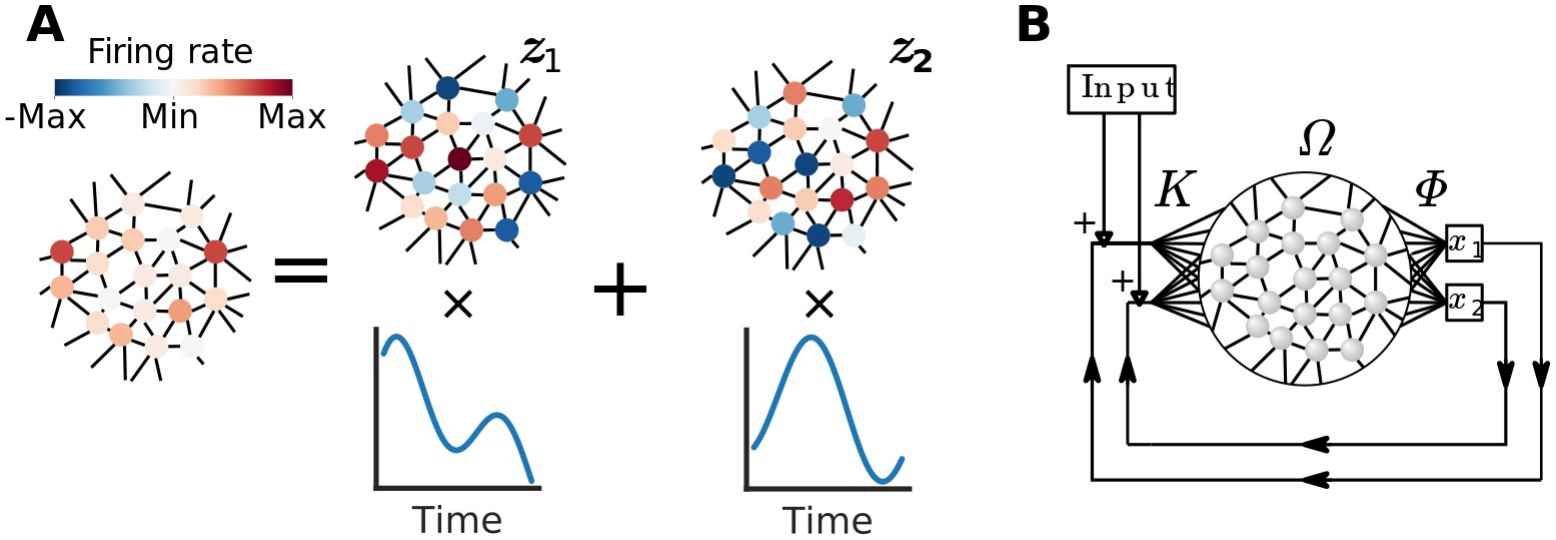
A schematic of the architecture for creating neural modes. (**A**) Low dimensional neural activity can be decomposed into neural modes (for illustration here we consider two neural modes: **z**_1_ and **z**_2_). (**B**) The neural architecture shared among all three frameworks used in this work. A “reservoir” of neurons that are connected with synaptic weights Ω_*ij*_ generates some activity. From this activity *D* = 2 latent variables (*x*_1_ and *x*_2_) are read out using the weights Φ. The values are then fed back into the network using weights *K*. Note that *K* and Φ do not represent any physical entities, but only serves as helper matrices to eventually construct the weight matrix *W* (see Eq 5).

Neural activity with *D* dimensions can by definition be decomposed into *D* time-independent components called principal components, factor loadings or *neural modes* (Fig 1A). The vector of instantaneous firing rates **a**(*t*) can at any point in time be described as a linear combination of these neural modes **z**_1_, **z**_2_, …, **z**_*D*_ plus some noise *ϵ*(*t*):

$$a\left(t\right)=\sum_{i=1}^{D}x_{i}\left(t\right)z_{i}+\epsilon \left(t\right)=Zx\left(t\right)+\epsilon \left(t\right)$$ (1)

The time-varying coefficients *x*_*i*_(*t*) carry computationally relevant information and are therefore referred to as *latent variables* [9]. In this work, we measure the **a**(*t*) as the number of spikes per consecutive 50ms-bin. However, note that **a**(*t*) is intuitively similar to the filtered spike trains **u**(*t*) that are defined below.

To explain the relation between neural modes and learning discovered by Sadtler et al. [4] we need to construct a network in which we can control the neural modes and the latent variables. We do this by beginning to model the network as a simple memory circuit (Fig 1B): once presented with some continuous variable, the network should retain this variable in its intrinsic activity so that it can be read out later. We argue that the ability to robustly encode variables in this fashion is analogous to the ability to keep a BCI readout stable. Nevertheless, some BCI tasks may require the readout to not only be stable and static, but dynamic. Therefore, we have structured the rest of the Results as follows: first we show that synaptic weights are similar before and after an inside-manifold perturbation in networks with static latent variables. Second, we show that this result extends to some degree to networks where the latent variables oscillates. Finally, we show that it also holds for a more biologically plausible network.

### Model

We consider networks of *N* leaky integrate-and-fire (LIF) neurons. The sub-threshold membrane voltage for neuron *j* is given by

$$\tau_{m}\frac{dV_{j}}{dt}=\left(V_{\text{leak}}-V_{j}\right)+R_{m}I_{j}$$ (2)

where *τ*_*m*_ = *C*_*m*_*R*_*m*_ is the membrane time constant, *C*_*m*_ is the membrane capacitance, *R*_*m*_ is the membrane input resistance and *I*_*j*_ is the total time-varying synaptic and external input current. Once the membrane voltage passes a threshold *V*_th_ a spike is emitted and the membrane voltage is reset to *V*_reset_.

The challenge is to connect the synapses of the network is such a way so that the network becomes a (*D*-dimensional) line attractor capable of retaining the value of *D* latent variables. That is, how should the network be connected so that the input currents *I*_*j*_ (synaptic or otherwise) to each neuron contributes to keeping the latent variables stable?

The general strategy we use for doing this is illustrated in Fig 1B. For each latent variable we create a corresponding latent readout-node that decodes the variable from the network activity by taking a linear combination of the synaptically filtered spike trains:

x(t)=Φu(t) (3)

where $\Phi \in R^{D\times N}$ is the set of weights, *u*_*j*_(*t*) = *H* * ∑_*m*_ *δ*(*t*_*jm*_ − *t*) are the filtered spike trains, *H* is a synaptic kernel and *t*_*jm*_ is the time of the *m*th spike from neuron *j*.

We can use the read out latent variables **x** to construct an input current to the neurons that feeds the latent variables back into the network:

$$I_{\text{fb}}=Kx\left(t\right)$$ (4)

where $K\in R^{N\times D}$ is an arbitrarily chosen matrix (Fig 1B) and **I**_fb_ is vector notation for one part of the synaptic currents to the neurons (Eq 2). This artificial construct can in turn be used to find a set of synaptic weights: note that the decoding (Eq 3) and the feed-back (Eq 4) steps are both linear operations, and can thus be combined into one single matrix multiplication

$$I_{\text{fb}}=K\Phi u\left(t\right)=Wu\left(t\right)$$ (5)

From this, we identify $W\in R^{N\times N}$ as a matrix of synaptic weights and a tentative solution to the problem of how to connect the network (for a more detailed derivation of this result, see Abbott et al. [10]).

In particular, a network connected in this fashion has a basic structure needed for retaining *D* latent variables once they have been set. However, several more details have to be determined before implementing this idea in practice. The most crucial issue is how to find the decoding weights Φ in Eq 3. Moreover, we need to determine whether any additional synapses are required in addition to the ones indicated by *W* in Eq 5. In this paper, we consider three different strategies for finding Φ and designing the connections.

First, we consider the FORCE learning rule [12] which builds upon the liquid state machine and echo state networks [11, 17, 18]. Following this idea, the synapses of the network belong to two sets. The synapses of the first set are chosen randomly, but are normalized so that the input to each neuron is loosely balanced, which makes the network dynamics chaotic [19]. The second set of synapses directly corresponds to the matrix *W* above. Thus, for FORCE networks, the current in Eq 2 is given by

$$I\left(t\right)=\underset{I_{\text{res}}}{\underset{︸}{\Omega u(t)}}+\underset{I_{\text{fb}}}{\underset{︸}{Wu(t)}}$$ (6)

where $\Omega \in R^{N\times N}$ is the matrix corresponding to the first set synapses and *W* = *K*Φ correspond to the second set as above. The first matrix Ω and the encoder weights *K* are both fixed while Φ are subject to an online learning rule (see [12] and Methods).

Second, we consider the Neural Engineering Framework (NEF) [13, 14]. Unlike the FORCE algorithm, NEF does not require any extra synapses in addition to ones implied by *W*. Instead, each neuron receives an additional stationary driving current so that the total current becomes

$$I\left(t\right)=Wu\left(t\right)+I_{\text{drive}}$$ (7)

Similar to the FORCE rule, in the NEF *W* = *K*Φ where *K* is fixed and Φ is learned. In the NEF however, Φ is learned in batch. That is,

$$\Phi =\underset{\Phi^{\prime }}{\text{arg}\;\text{min}}\langle ∥x_{\text{target}}-\Phi^{\prime }u{∥}^{2}\rangle_{x_{\text{target}}}$$ (8)

where the expected value is taken over all permitted values of **x**_target_ and the corresponding filtered spike trains **u** (see [13] and Methods).

Finally, we consider the Efficient Coding framework [15, 16]. Similar to the FORCE learning, there are two sets of synapses, a set for the reservoir and another set for the recurrent connections. In contrast to FORCE learning and the NEF, no learning is required in the Efficient Coding framework. Instead, the readout weights are fixed to

$$\Phi =\lambda_{\text{syn}}K^{T}$$ (9)

where λ_syn_ = 1/*τ*_syn_. At the same time, the reservoir weights are fixed to

$$\Omega =-KK^{T}-\mu \lambda_{\text{syn}}^{2}I$$ (10)

rather than being chosen randomly. Finally, the reservoir synapses are assumed to be instantaneous, i.e. they have a time constant of 0. The input current to neurons are given by

$$I\left(t\right)=\Omega u_{\tau =0}\left(t\right)+Wu\left(t\right)$$ (11)

where *W* = λ_syn_ *KK*^*T*^, and **u**_*τ* = 0_(*t*) are unfiltered spike trains.

### The neural modes and the decoders are both determined by the encoders

**Fig 2:**
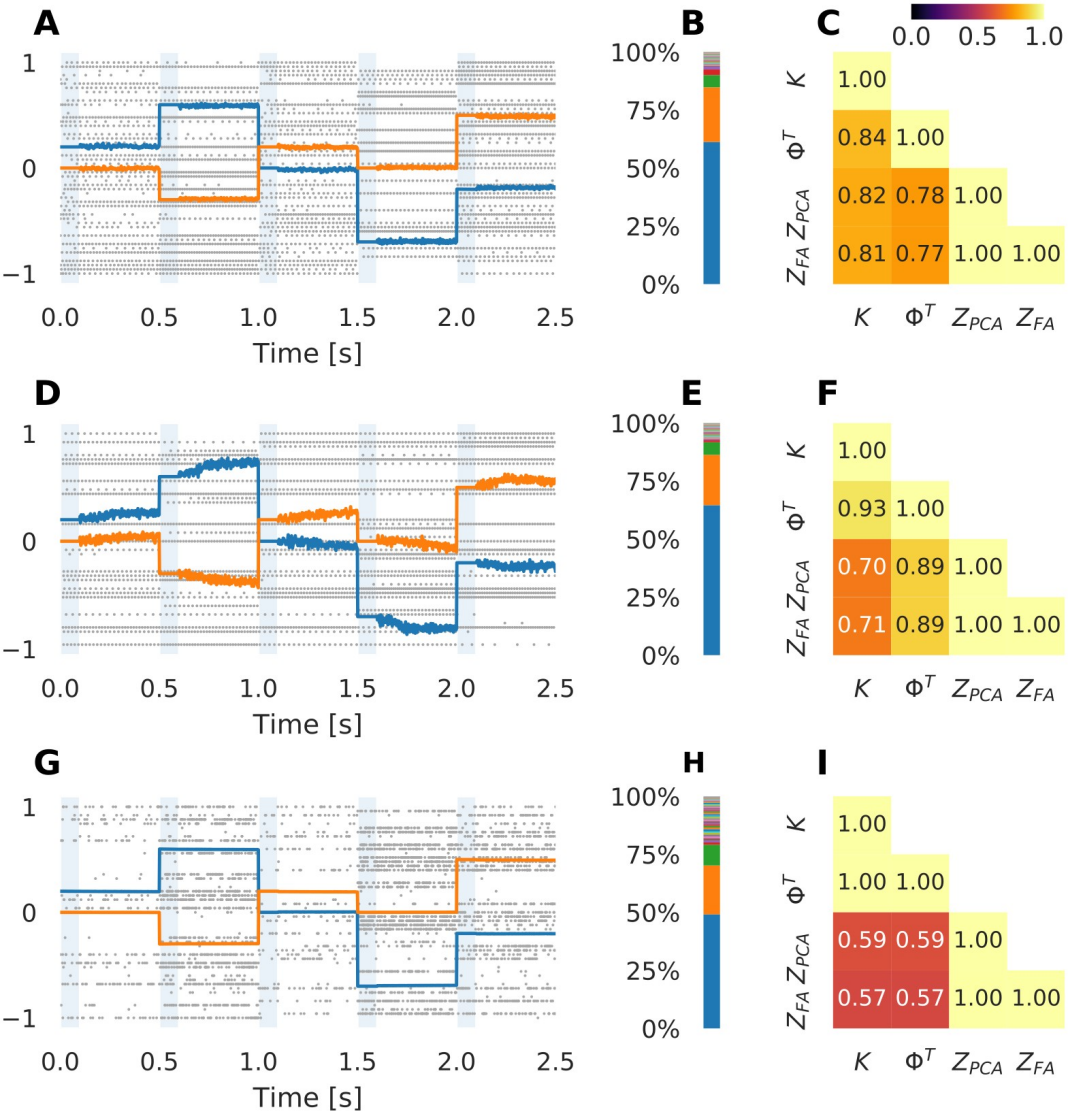
The encoders *K* determine the decoders Φ^*T*^ and the neural modes *Z*. (**A**) Example of a simulation of a spiking network with the FORCE learning rule. The two traces show the two latent variables. During the periods shaded blue, the latent variables are fixed. The spike times of 50 (5% of the) neurons are shown in the background. (**B**) The fraction of spike rate variance explained by each principal component. (**C**) The generalized correlation (see Methods) between the encoder matrix *K*, decoder matrix Φ^*T*^, principal components *Z*_PCA_ and loading matrix *Z*_FA_. (**D, E, F**) Same result for the Neural Engineering Framework. (**G, H, I**) Same result for the Efficient Coding framework.

In Fig 2 we demonstrate the ability of the three frameworks to retain a value once it has been set. We implement a network with *N* = 1000 neurons and *D* = 2 latent variables for each of the three frameworks mentioned above. We verify the implementations using the following protocol: every 500 ms we artificially fix the two latent variables *x*_1_ and *x*_2_ to some arbitrary values during 100 ms (blue areas in Fig 2A, 2D and 2G) and then let the activity evolve freely for the next 400 ms. As intended from the design of the networks, they retain the variables once they have been set, albeit with varying degrees of noise and drift.

For all three frameworks, the spiking pattern is largely determined by the latent variables. In particular, note that the spiking pattern (background of Fig 2A, 2D and 2G) changes considerably when the values of the latent variables are changed. These changes in firing pattern are due to the divergent connection *K* from the latent variables **x** to the input currents **I** (see Eq 4 and Fig 1B), which are created so that each neuron receives an input that is a linear combination of the latent variables. We expect the firing rates to approximately reflect these input currents, albeit with some distortion due to auxiliary synapses, threshold nonlinearity in the neuron and binning to estimate the firing rate.

To verify this, we estimated the firing rate in Fig 2A, 2D and 2G by counting the number of spikes from each neuron in each consecutive 50 ms interval. We then applied Principal Component Analysis (PCA) on the time series of spike bins. As can be seen in Fig 2B, 2E and 2H for all our networks two principal components explained the majority of the variance. We then calculated the cosine of the mean principal angle between the subspace spanned by the first two principal components *Z*_PCA_ and the subspace spanned by the columns of the encoder matrix *K* (this is a generalization of correlation, see Methods) and found a high degree of similarity (cos *ϕ* ≈ 0.7) for all three frameworks (Fig 2C, 2F and 2I). We also observed a similar degree of similarity with the loading matrix *Z*_FA_ from Factor Analysis (FA)—indeed, the subspaces found by FA were almost completely equal to the the subspaces found by PCA. Equating the principal components with neural modes we conclude that neural modes are determined by the encoder matrix *K*. Note that all the three frameworks allow us to choose *K* arbitrarily. Thus, they provide a way to construct networks where we can freely select not only the number of neural modes (i.e. dimensionality), but also the specific mode vectors.

The fact that the neural activity is determined by the neural modes further influences the readout weights Φ. Although the specific algorithm for determining Φ differs between the three frameworks, they all have in common that Φ should be an accurate linear transform from the spike trains to the latent variables. If the neural modes *Z* are orthonormal, a latent variable can be read out by taking the inner product between the respective neural mode and the neural activity: *x*_*i*_ ≈ **z**_*i*_ ⋅ **u**. Therefore, we expect the readout weights to be Φ^*T*^ ≈ *Z* (where the columns of *Z* span the same space as *Z*_PCA_ and *Z*_FA_). For a more formal treatment of this argument, see Salinas and Abbott [20].

Given that Φ^*T*^ ≈ *Z* and *Z* ≈ *K* it is safe to assume from transitivity that

$$\Phi^{T}\approx K$$ (12)

For the three frameworks under consideration here the correlation between Φ^*T*^ and *K* is in fact even larger than between *Z* and *K* or between *Z* and Φ^*T*^ (Fig 2C, 2F and 2I). For FORCE learning and the NEF, this is likely because while *Z* is found to be the directions of maximal variance, Φ^*T*^ is found by supervised learning so that the readout match the “real” latent variables as close as possible. For Efficient Coding, the correlation between *K* and Φ^*T*^ is exactly 1 because of Eq 9.

### Synaptic modifications required for inside- and outside-manifold perturbations

Now we use the previous networks to address the main issue of this paper: why are some perturbations of the neural modes much easier to learn than others? We know that the neural modes *Z* ≈ *K* for the type of networks we are studying here. Thus, if *Z* is to be perturbed to perform the task, a corresponding perturbation must be applied to the encoders *K*.

Sadtler et al. [4] characterized perturbations of the neural modes into *inside-manifold* perturbations and *outside-manifold* perturbations. An inside-manifold perturbation is restricted so that the subspace spanned by the neural modes (the *intrinsic manifold*) is preserved. In other words, even though the neural modes have been changed, the part of the neural state space that is allowed is unchanged. Mathematically, the perturbed neural modes of an inside-manifold perturbation are given by

$$\tilde{Z}=Z\tilde{Q}$$ (13)

where $\tilde{Q}\in R^{D\times D}$. Here, we assume that the inside-manifold perturbation is defined to not stretch or skew the subspace, and therefore that $\tilde{Q}$ is an orthogonal matrix. We refer the treatment of non-orthogonal matrices to S1 Text.

Assuming the *Z* ≈ *K* ≈ Φ^*T*^ holds for any network, it will also hold for the permuted network, i.e. $\tilde{Z}\approx \tilde{K}\approx {\tilde{\Phi }}^{T}$, we can expect the corresponding changes to encoder and readout matrices to be

$$\tilde{K}\approx K\tilde{Q}\;\text{and}\;{\tilde{\Phi }}^{T}\approx \Phi^{T}\tilde{Q}$$ (14)

For all three frameworks the set of (learnable) synaptic weights are given by *W* = *K*Φ. Thus, the new set of synaptic weights after the perturbation is:

$$\tilde{W}=\tilde{K}\tilde{\Phi }\approx K\tilde{Q}\left({\tilde{Q}}^{T}\Phi \right)=K\Phi =W$$ (15)

where $\tilde{Q}{\tilde{Q}}^{T}=I$ follows from orthogonality. Changing the synaptic weights from *W* to $\tilde{W}$ would therefore only require minor modifications, if any.

This result does not hold for outside-manifold perturbations. Such a perturbation can be written as

$$\hat{Z}=\hat{Q}Z$$ (16)

where $\hat{Q}\in R^{N\times N}$ is also an orthogonal matrix, although with many more elements than $\tilde{Q}$ (*N*^2^ vs *D*^2^). Following the same steps as above, one arrives at

$$\hat{W}\approx \hat{Q}K\Phi {\hat{Q}}^{T}=\hat{Q}W{\hat{Q}}^{T}$$ (17)

In general, $\hat{W}$ is not equal to *W* and therefore learning $\hat{W}$ likely requires substantial synaptic changes and hence extensive learning.

To verify this derivation, we created three new networks similar to the ones in Fig 2. Keeping all other parameters equal, we permuted the two columns of *K* to mimic inside-manifold perturbations, i.e. we applied Eq 14 with

$$\tilde{Q}=[\begin{array}{cc} 0 & 1\\ 1 & 0 \end{array}]$$ (18)

**Fig 3:**
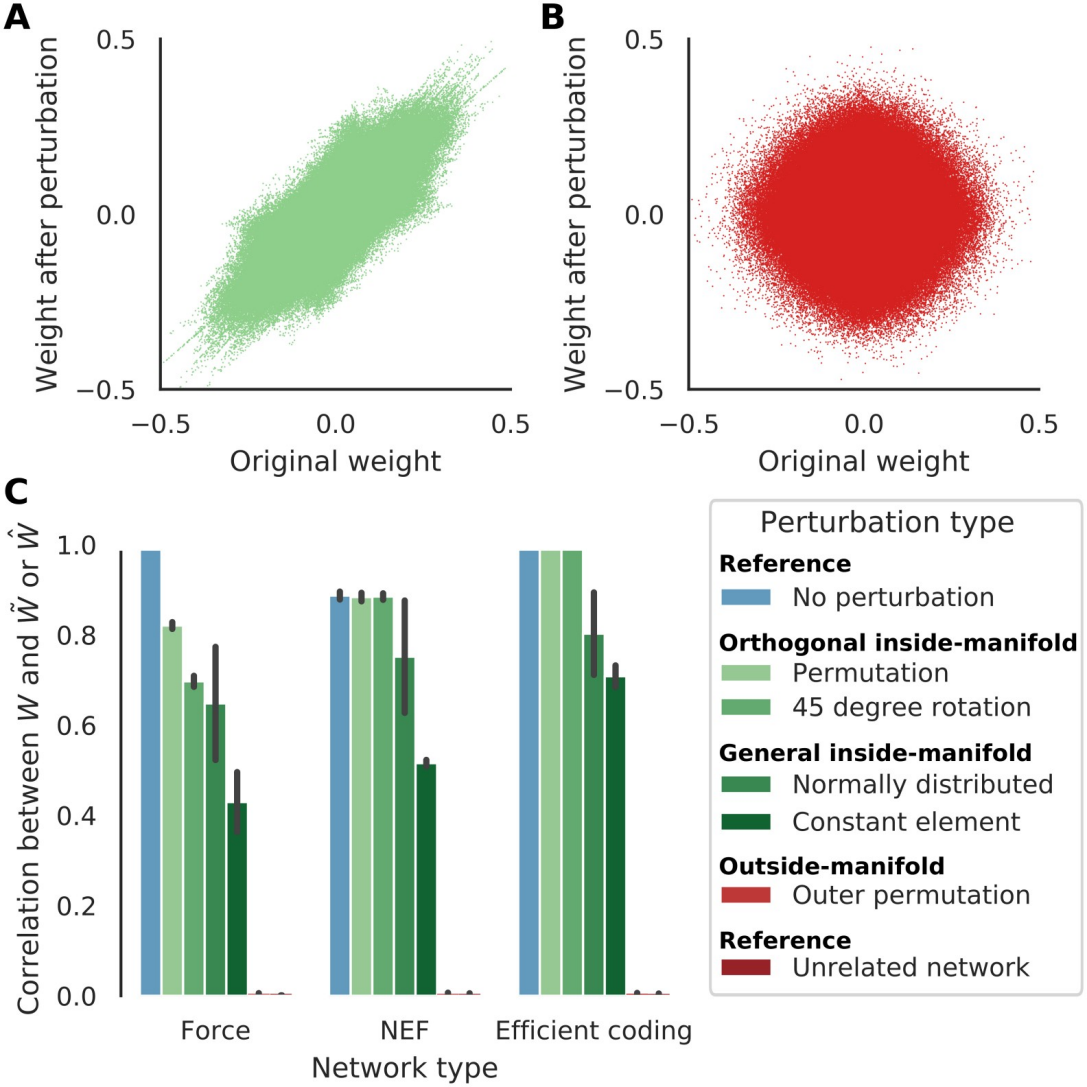
Inside manifold perturbations do not require big changes of synapse weights. (**A**) Synapse weights (elements of *W*) from one network instantiation of FORCE, compared to re-learning with the permuted encoders $\tilde{K}$ (elements of $\tilde{W}$). (**B**) Same as A, but for an outside-manifold permutation ($\hat{W}$). (**C**) The element-wise correlations between old (*W*) and new synaptic weights for different types of permutations (see main text for definitions) for all three frameworks. Error bars indicate standard deviation over 30 network instantiations. Note that orthogonal inside manifold perturbations (“Permutation” and “45 degree rotation”) require almost no changes to the weights, non-orthogonal inside manifold perturbations (“Gaussian elements” and “Same element”) require some changes and the outside-manifold permutation requires as much change as a network with unrelated encoders. See also S1D Fig.

We then learned $\tilde{\Phi }$ using the respective framework, and calculated $\tilde{W}=\tilde{K}\tilde{\Phi }$. For comparison, we created an additional three networks with the same parameters as before, but where the rows of *K* were permuted by switching the first 500 rows with the last 500. That is, we used

$$\hat{Q}=[\begin{array}{cccccc} 0 & 0 & \cdots & 1 & 0 & \cdots \\ 0 & 0 & \cdots & 0 & 1 & \cdots \\ \vdots & \vdots & \ddots & \vdots & \vdots & \ddots \\ 1 & 0 & \cdots & 0 & 0 & \cdots \\ 0 & 1 & \cdots & 0 & 0 & \cdots \\ \vdots & \vdots & \ddots & \vdots & \vdots & \ddots \end{array}]$$ (19)

to mimic outside-manifold transformation and learned $\hat{\Phi }$ and $\hat{W}=\hat{K}\hat{\Phi }$ as before. We thus have examples of *W*, $\tilde{W}$ and $\hat{W}$ for the three frameworks. In Fig 3, we show that the element-wise correlation is high between *W* and $\tilde{W}$ (inside-manifold permutation, light green) but close to zero for *W* and $\hat{W}$ (outside-manifold permutation, red). A high correlation indicate that only small changes to synaptic weights are required to change the neural modes within the intrinsic-manifold suggesting that this change may be easier for a biological network to learn. Note that correlations do not capture global additive or multiplicative scaling of the weights. Given that the networks have otherwise identical parameters before and after perturbation, we have no reason to expect any such scaling their weights. Nevertheless, to avoid this possibility, we also calculated the Frobenius norm which also showed a very similar pattern (S1 Fig).

For further comparisons, we introduced four additional inside manifold perturbations: 1. no perturbation at all, just restarting the learning algorithm with the same *K* (Fig 3C, light blue), 2. an orthogonal inside manifold perturbation given by the following transformation (Fig 3C, green):

$${\tilde{Q}}_{\text{rot}}=[\begin{array}{cc} \text{cos}\frac{\pi }{4} & -\text{sin}\frac{\pi }{4}\\ \text{sin}\frac{\pi }{4} & \text{cos}\frac{\pi }{4} \end{array}]$$ (20)

3. a non-orthogonal perturbation matrix ${\tilde{Q}}_{\text{gaussian}}$ where all four elements were independently drawn from N(0,1) (Fig 3C, dark green), a non-orthogonal matrix ${\tilde{Q}}_{\text{same}}$ where all four elements were equal (to a random value) (Fig 3C, darker green). In each of these different types of inside-manifold perturbations, element-wise correlation between *W* and $\tilde{W}$ was high, further suggesting that inside manifold perturbation required relatively small change in synaptic weights. By contrast, correlation between $\tilde{W}$ corresponding to a network where *K* was redrawn independently and *W* was almost zero (Fig 3C, dark red). These results were independent of the way we measured the similarity between *W* and $\tilde{W}$ (S1 Fig).

Note that in the frameworks we have used here the learning rule is applied to Φ and only indirectly to *W*. In general Φ is not correlated to either $\tilde{\Phi }$ or $\hat{\Phi }$ (see S1A, S1B and S1C Fig). The correlation only becomes visible after multiplying *K* and Φ (see Eq 15). Furthermore, note that we have excluded the reservoir weights Ω from the correlation because we model them as being static. For Efficient Coding, we have ignored changes to the fast weights (Ω) because they are just replicates of the regular weights *W*.

### Manifold perturbations with dynamical latent variables

In Fig 2 we considered latent variables that were assigned values by some unspecified external control, and that once set the networks’ sole task was to retain them. In reality the functional role of most local circuits is doubtlessly more complex and likely involves manipulating the latent variables in relation to the inputs.

**Fig 4:**
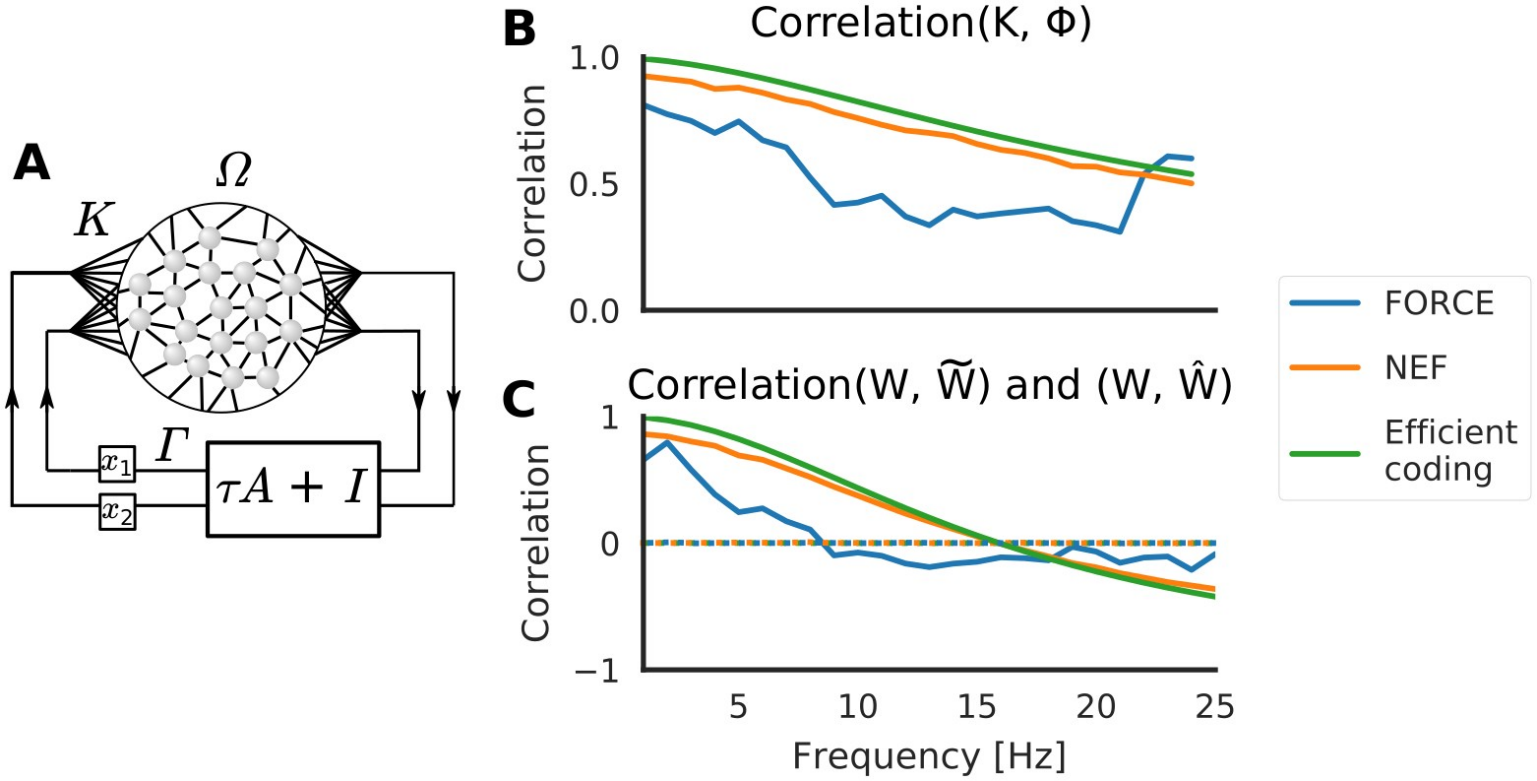
Oscillating latent variables. (**A**) By slightly modifying the architecture of Fig 1B, the network can be constructed to perform some computation on the latent variables, for example evolving them as a linear dynamical system (Eq 22). (**B**) The element-wise correlation between *K* and Γ for increasing frequencies *f* = *ω*/2*π*. (**C**) The element-wise correlation between synaptic weights before and after an inside-manifold perturbation (permuting the columns of *K*, solid lines), and before and after and outside-manifold perturbation (permuting the rows, dotted lines).

The static attractor model can easily be extended to allow for some computations on the latent variables. In particular, we can replace the decoder weights Φ with some other weights *Γ* and choose these so that the latent variables are modified before they are fed back into the network (Fig 4A). Notably, if choose

$$\Gamma =(\tau_{\text{syn}}A+I)\Phi$$ (21)

where $A\in R^{D\times D}$, the dynamical variables will evolve according to

$$\frac{dx}{dt}=Ax$$ (22)

In the Efficient coding framework, Eq 21 is explicitly applied to find the new weights Γ. In FORCE and the NEF however, the error function of the learning is changed so that Eq 21 will only be applied implicitly. Finding Γ using this implicit method has the benefit that Γ can represent both linear and non-linear transformations of **x** [14], thus enabling more complex dynamics than suggested by Eq 21.

Here we first consider the linear case of two-dimensional oscillators, i.e.

$$A=[\begin{array}{cc} 0 & -\omega \\ \omega & 0 \end{array}]$$ (23)

There are three reasons we study oscillating dynamics in lieu of some more complex dynamical system: (1) it is straight-forward to implement comparable dynamics in all three frameworks, (2) the frequency can be continuously varied to demonstrate the difference between fast and slow dynamics, and (3) periodic and quasi-periodic movements are often observed in natural behavior and weakly periodic neural activity patterns have been observed in certain brain regions e.g. motor cortex [21].

Following Fig 2C, 2F and 2I, we measured the correlation between the encoders *K* and the decoders Γ for different oscillation frequencies to study slow and fast dynamics of the neural modes. We found that the correlation between *K* and Γ decreased with increasing oscillator frequency. We expect this effect because from Eqs 23 and 21 we have

Γ→Φwhenω→0 (24)

For the Efficient coding framework, Eq 21 is applied explicitly. Therefore, in Fig 4B the Efficient coding trace could also be viewed as the ideal correlation predicted by the theory. The NEF follows this closely, while the FORCE deviates a bit. In any case, seeing that Γ is correlated with *K* for low frequencies, we can conclude that even for dynamical latent-variables inside-manifold perturbations should require less change of the synaptic weights than outside-manifold perturbations. In Fig 4C we verify that this is indeed the case: for low frequencies the synaptic weights after an inside-manifold perturbation are highly correlated with the originals (solid lines) while the correlation is robustly (close to) 0 for outside-manifold perturbations. Thus, for low frequencies, only small changes of the synaptic weights are required to make an inside-manifold perturbation. The procedure for creating Fig 4B and 4C was very similar for creating figure Fig 3: the three networks consisted of *N* = 1000 neurons each, and inside and outside-manifold perturbations were given by Eqs 18 and 19, respectively.

Thus, we argue that introducing slow dynamics does not invalidate our claim that inside-manifold perturbations will be easier to learn. If the latent variables reflect motor behaviors, it is reasonable to assume that they vary with behavioral timescales (hundreds of milliseconds), i.e. less than 5-10 Hz. Indeed, the oscillations found by Churchland et al. [21] appears to be about 1 Hz. Note that oscillations of latent variables do not in general reflect oscillations of neural activity (*δ*, *α*, *β*, *γ*, etc.).

### Networks with sparse and Daleian connectivity

While the networks created up to this point exhibit many biologically plausible features, the matrices of synaptic weights *W* are dense and do not obey Dale’s law [22]. Because our claim is centered around the similarity between these matrices before and after the perturbations, it is necessary to verify that adding biological constraints does not nullify the result. Therefore, we here introduce network sparsity and Dale’s law.

We focus in this section on the Neural Engineering Framework because its use of explicit optimization makes it relatively straight-forward to introduce constraints. However, in the NEF (Eq 8) the variable being optimized is the decoders Φ (or, in the non-stationary case, Γ), and not the synaptic weights *W*. Therefore, to directly impose constraints on *W* we changed the optimization procedure to

$$W=\underset{\text{sign}(W^{\prime })=C}{\text{arg}\;\text{min}}\langle ∥Kf\left(x_{\text{target}}\right)-W^{\prime }u{∥}^{2}\rangle_{x_{\text{target}}}$$ (25)

where

$$\text{sign}\left(w_{ij}\right)=\{\begin{array}{cc} 1 & \text{if}\;w_{ij}>0\\ 0 & \text{if}\;w_{ij}=0\\ -1 & \text{if}\;w_{ij}<0 \end{array}$$ (26)

Thus, by appropriately choosing *C*, we can constrain *W*. We enforced sparsity and Dale’s law by setting

$$C_{ij}=\{\begin{array}{cc} 0 & \text{if}\;R_{ij}<\xi \\ 1 & \text{if}\;R_{ij}\geq \xi \;\text{and}\;j\leq \epsilon N\\ -1 & \text{if}\;R_{ij}\geq \xi \;\text{and}\;j>\epsilon N \end{array}$$ (27)

where *R*_*ij*_ is draw randomly from a uniform distribution between 0 and 1, *ξ* controls the sparsity and *ϵ* is the fraction of excitatory neurons. Also note that we have changed the target from **x**_target_ in Eq 8 to *f*(**x**_target_) for some function *f*(⋅) in Eq 25, so that temporal dynamics can be introduced.

We used Eq 25 to construct a network with *N* = 5000 neurons and *D* = 4 latent variables. The increase in network size helps to prevent the in-degrees from being too small given the sparser connectivity. The dynamics *f*(⋅) were chosen so that the first two latent variables would be the leading and lagging components of a 2 Hz oscillator and the last two would similarly be the lead and lag of a 4 Hz oscillator. Additionally, we took advantage of NEF’s possibility of non-linearities and added a term to *f*(⋅) causing the oscillation amplitude to converge to 1 (see Methods).

We chose *ϵ* = 0.8, corresponding to 80% excitatory neurons and 20% inhibitory neurons and matching the observed ratio of excitatory and inhibitory neurons in the neocortex [23]. To achieve plausible sparsity we set *ξ* = 0.75 meaning only 25% of the connections were allowed. However, even when *C*_*ij*_ ≠ 0 it is still allowed for *W*_*ij*_ to be arbitrarily close to 0. Excluding connections numerically indistinguishable from 0, the connection probability was 10.8% for excitatory neurons (*j* ≤ 4000) and 19.0% for inhibitory neurons (*j* > 4000). These connection probabilities are well within the biological ranges [24–26].

**Fig 5:**
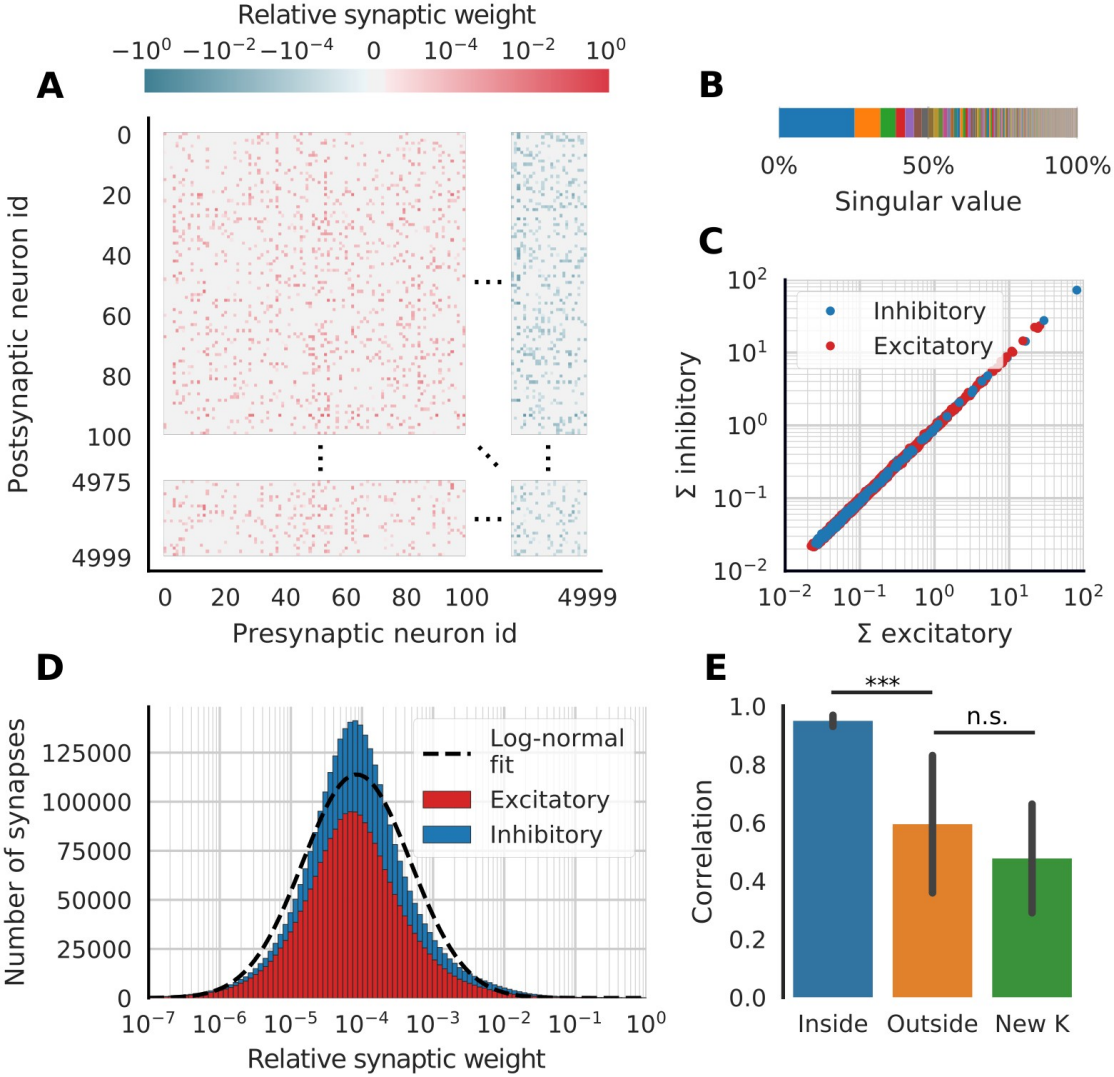
The biologically plausible weights matrix *W*. (**A**) The synaptic weight matrix is sparse and Daleian (neurons with id ≥ 4000 only make inhibitory connections). Only 100 excitatory and 25 inhibitory neurons are shown (**B**) Singular value decomposition of weight matrix *W*. *W* has more than four non-zero singular values, i.e. rank >*D*. (**C**) The sum of incoming excitatory weights to each neuron (one point) is similar to the sum of incoming inhibitory weights, indicating E/I balance. (**D**) Distribution of synaptic weights. The distribution is heavy-tailed but not perfectly log-normal. (**E**) Correlation between *W* and $\tilde{W}$ (inside manifold perturbation of *K*) and between *W* and $\hat{W}$ (outside manifold perturbation). For comparison a third option with a completely independent *K* is also shown. Error bars indicate standard deviation over 23 different permutations.

The matrix *W* is shown in Fig 5A and exhibits many biologically plausible features in addition to being Daleian and sparse. For instance, the sum of all excitatory inputs matches the sum of all inhibitory inputs to every neuron (Fig 5C). Furthermore, the synaptic weights have a heavy-tail distribution (Fig 5D). Although a closer analysis reveals it to be somewhat leptokurtic (kurtosis is 4.56 for logarithmized weights), the weights distribution is in any case rather close to being log-normal across more than five orders of magnitude. This conforms with recent experimental data suggesting that the weight distributions in biological neural networks are typically heavy-tailed, if not perfectly log-normal [27].

We then exposed this model to the same type of perturbations of the encoders *K* as we did for the previous models. However, while only one non-identity permutation matrix exists for *D* = 2, there are 4! − 1 = 23 non-identity permutations for *D* = 4. Still restricting our choice of perturbations to permutations, there are hence 23 possibilities for $\tilde{Q}$. We calculated the element-wise correlation between *W* and $\tilde{W}$ for all 23 perturbations. Similarly, we generalized Eq 19 to 4-fold permutations and calculated the element-wise correlation between *W* and $\hat{W}$ for all 23 non-identity permutations. The correlations for inside-manifold perturbations were significantly higher than outside-manifold perturbations (Fig 5E, *p* = 6.1 ⋅ 10^−7^, dependent samples t-test). Note that the same *C* was used for all weight matrices and that this incurs some structure that increases the correlation. To assess the magnitude of this effect, we redrew 23 independent encoder matrices *K*′, computed the corresponding weight matrices and correlated them with *W*. These correlations were not significantly different than the outside-manifold perturbations (Fig 5E, *p* = 0.12, dependent samples t-test). In other words: although outside-manifold perturbations evince non-zero correlations, they still require changing the synaptic weights almost as much as when learning a completely new set of neural modes.

## Discussion

Low-dimensionality has recently emerged as a prominent feature of the neuronal activity in the neocortex [4, 28, 29]. There is a growing interest in understanding the origin and maintenance of the low-dimensional activity. Several studies have proposed mechanisms and constrains necessary to generate low-dimensional activity in recurrent neural networks [5, 6, 30, 31]. An important feature of the low-dimensional activity of the neurons is that animals find it very hard to generate activity that lies outside the intrinsic manifold [4]. In this article, for the first time, we provide an explanation of this experimental observation.

We used three popular frameworks to design functional networks—Liquid State Machines with FORCE learning, the Neural Engineering Framework and Efficient Coding networks. First we demonstrate that there is a close similarity between the neural modes and a parameter matrix here called the *encoders* (*K*). Assuming the brain employs something akin to these frameworks, we argue that altering the neural modes (*Z*) would require changing the encoders (*K*). The encoders, in turn, are related to the recurrent connectivity within the network (*W*) in such a manner that certain large changes to the encoders do not require similarly large changes of the synaptic weights. This type of changes corresponds to *inside-manifold perturbations* if we follow the terminology used by Sadtler et al. [4]. By contrast, *outside-manifold* perturbations require large changes in the recurrent synaptic weights. Because learning is commonly believed to be implemented in terms of synaptic plasticity, our results provide an explanation of the difference in learning difficulty for the two types of perturbations as reported by Sadtler et al. [4].

Whether the brain does employ anything like these frameworks remains an open question. However, these frameworks provide more insights about the nature of low-dimensional activity and learning difficulty than other competing models of emergence of neural modes. For instance, although a clustered architecture also results in low dimensional activity [5, 6], clusters are not consistent with direct measurements of neural activity [6]. Alternatively, one can argue that neural modes could be a consequence of feed-forward connectivity. This would certainly be possible for circuits with strong, low-dimensional inputs and weak or absent lateral connections. However, this merely transfers the problem of creating a low dimensional signal to the afferent circuit. Solving this problem requires structured connections at some stage. The strength of the models we have used is precisely that they intrinsically ensures low-dimensional activity by implicitly creating a subspace attractor pulling the activity back to the space of neural modes [14]. In other words, components of the input orthogonal to this subspace are rapidly canceled out.

On the practical side, the architecture is particularly useful for studying neural modes in spiking networks because by using the frameworks described in this article the experimenter is free to explicitly specify any number of latent variables, any particular neural modes and any dynamics of the latent variables. For this purpose, it is important to emphasize that the architecture and the frameworks should be seen as recipes for specifying the synaptic weights and not as anatomical predictions: the readout nodes of Fig 1B do not reflect any biological entity, and the helper matrices *K* and Φ do not exist other than as components of the matrix of synaptic weights *W*. This distinction is also crucial for our results: the three frameworks in their simple forms optimize Φ and not *W*, yet our prediction is only that *W* will be similar before and after an inside-manifold perturbation. In fact, Φ and $\tilde{\Phi }$ will in general be uncorrelated, which makes it difficult to estimate learning time or convergence rates.

One might expect the matrix of synaptic weights *W* for *any* network with neural modes to be decomposable into two matrices *K* and Φ [7]. However, such decomposition can only be done when *W* has rank ≤*D* and as we show in Fig 5B, it is possible to construct a network with neural modes but with a non-decomposable weight matrix. Lifting the requirement that the weight matrix must be decomposable is likely favorable when extending this work to networks with a heterogeneous populations of neurons and synapses (for example, it is difficult to decompose a weight matrix if each synapse not only has a different weight but also different time constant). Recently Mastrogiuseppe and Ostojic [31] have argued that dense low rank matrices give low dimensional activity. Here, we show that this is not a necessary condition and that balanced and sparse weight matrices can also give rise to low dimensional activity.

Here, we have limited our scope to study a local network in isolation. While sufficient for comparing inside and outside manifold perturbations, this limitation masks the fact that the latent variables are most likely evolving not only as a result of local dynamics (*A* in Eq 21 and *f*(⋅) in Eq 25), but also due to structured inputs (Fig 1B). Indeed, in Fig 2, we have exaggerated the strength and accuracy of inputs and postulated that they are strong enough to instantly overwrite the currently encoded variables. In reality, this is most likely not the case. Rather, we expect the time evolution of the latent variables to be a consequence of a deliberate combination of afferent inputs and local feedback (see [32] for an example of how a large set of local NEF-circuits can be connected to a functional whole).

The fact that latent variable dynamics can arise from two sources makes it precarious to draw conclusions from Fig 4. For example, it is tempting to assume that inside-manifold perturbations would also be hard to learn during tasks involving high-frequency motions. However, this is not necessarily the case because even in an integrator network it is possible for the latent variables to oscillate if the inputs are oscillating (although they will be low-pass filtered). The prediction of our model is therefore that the difference between inside- and outside-manifold perturbations will be more pronounced in networks where the *local* dynamics imposed on latent variables are slow or stationary, and vanish completely for networks with *intrinsically* rapidly oscillating latent variables (Fig 4). It would be interesting to measure the spectra of the latent variables (and not that of the neuronal activity) to know whether the cortical networks operate in the high or low frequency regimes (Fig 4). Tentative evidence suggests it is in the low frequency range (about 1 Hz) [21].

The presence of structured inputs raises the question: what really changes in the network when an inside-manifold perturbation is applied? Consider the networks in Fig 2. They are designed to have two neural modes and two latent variables. The dynamical equations of the two latent variables are however identical (integrate the inputs) and therefore, once a network has been created, the numbering of neural modes becomes arbitrary. PCA provides one way of assigning order by considering the variance explained be each neural mode, which in turn depends on the variance of the corresponding latent variable. In an integrator network the variance of each latent variable is completely determined by the inputs. Thus, for integrator networks, a permutation of the neural modes becomes equivalent to a permutation of the inputs. Indeed, when *Q* is a permutation matrix, Eq 15 states that changing the order of the neural modes does not imply *any* changes in the weight matrix (in the ideal case, or with Efficient Coding)—the only thing changing is the inputs.

**Fig 6:**
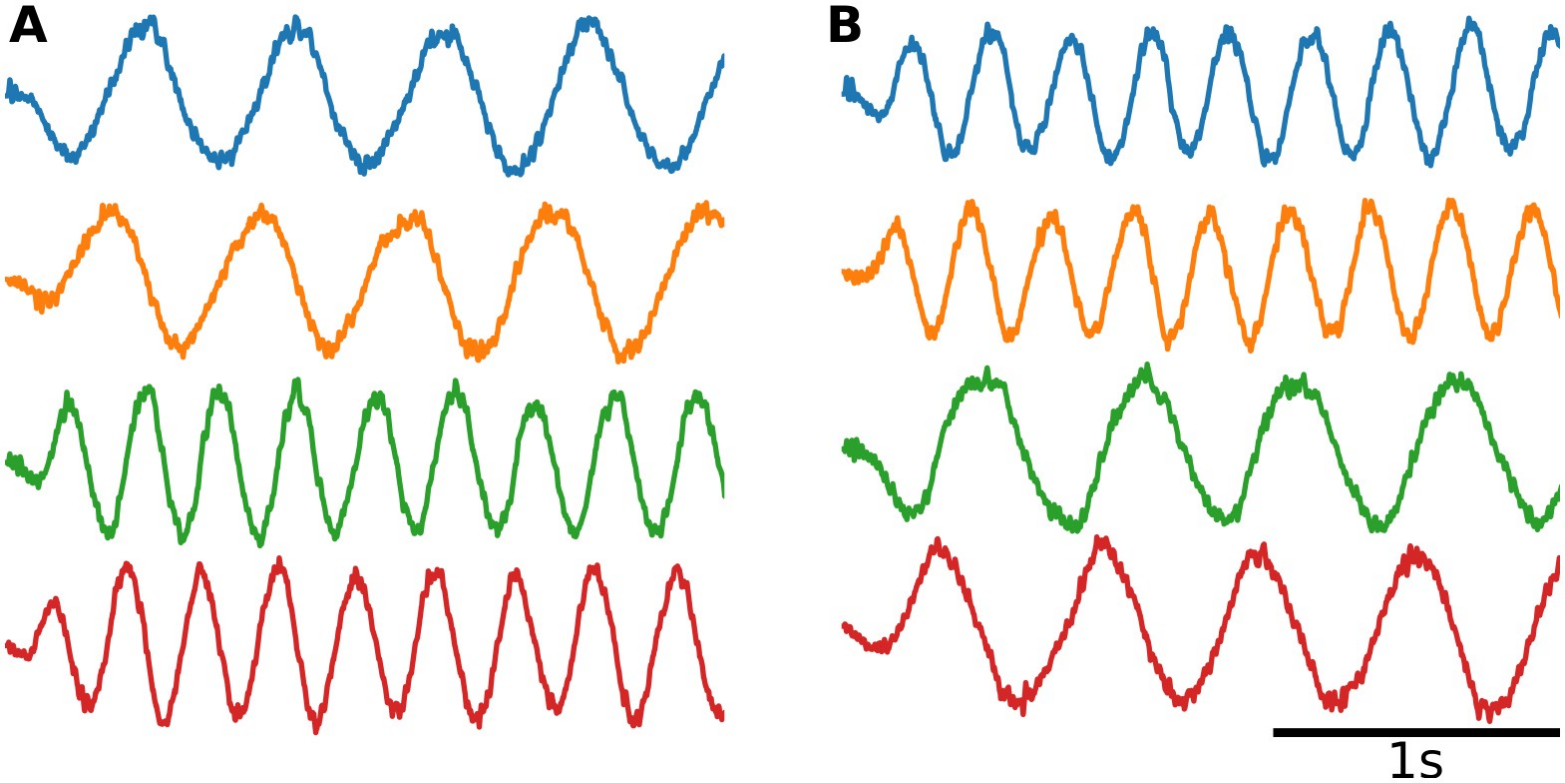
A permutation of the columns of *K* results in a permutation of the latent variables. (**A**) A readout of the four latent variables of the unperturbed network shown in Fig 5. (**B**) A readout of the latent variables of one of the perturbed networks (inside manifold permutation 3 4 1 2) using the readout weights from the unperturbed network, which results in a corresponding permutation of the latent variables. Note that this network receives no structured inputs and that the permutation of the latent variables is completely due changes in the local connections.

To verify that an inside manifold perturbation really does change something meaningful in the local network in a more general setting, one has to consider networks where the dynamical equations are not identical for all latent variables. For example, the latent variables of the network described in Fig 5 do have non-identical dynamical equations (Eq 35). In such a network we can directly read how inside manifold perturbation alters the latent variable calculated using the *same* decoding weights for the unperturbed and perturbed networks (compare Fig 6A and 6B). It is evident that the order of the latent variables have changed accordingly. Note that there are no structured inputs to this network and the permutation of the latent variables in this case stems completely from changing the local weights of the network. If we allow the readout weights abstractly represent the coefficients of the BCI interface as was done in Sadtler et al. [4], Fig 6 shows an example of how a modification to the local circuitry can alter the BCI readout in a systematic way. Recently, Golub et al. [33] provided some evidence that learning of inside-manifold perturbations may indeed be achieved by reassociation of latent variables with neural modes. With our results we argue that reassociation of latent modes may involves small changes in the network connectivity. It is also possible that the input to the network are altered to achieve inside-manifold perturbations [34]. However, changes the input requires changes in the manifold of the activity of an upstream network.

Although we have suggested one possible explanation of the findings of Sadtler et al. [4], our models are not intended to faithfully reproduce all aspects of their experiment. For example, in our simulations we use small networks that do not match the number of neurons in primate motor cortex, and we have simplified the intrinsic manifolds to be 2- or 4-dimensional while the recorded manifolds had around 10 dimensions. The conceptual result however holds for larger manifolds as well (as shown in S3A Fig for an NEF network). Another discrepancy with the experiments is that extracellular field recordings necessarily only sample a small subset of neurons in the network. By contrast, in our networks we have estimated the dimensionality using all the neurons. Subsampling of neurons does not affect our results, as long as the sampling of neurons represent an independent and identically distributed sample of the network and are independent with respect to the neural modes (as shown in S3B Fig for NEF network). However, spatial and cell-type inhomogenities (as is often the case with the networks in the brain) might lead to a dependent sampling so that the intrinsic manifold seen by experimenters does not fully match the true one of the network.

We have demonstrated that the difference between inside and outside manifold perturbations is independent of the choice of framework by repeating most simulations in the three frameworks. However, our results should not be considered a fair comparison between the performance of the three frameworks because the parameters and setup were slightly different between the frameworks (see Methods). Furthermore, for simplicity we used comparatively simple versions of the frameworks. For example we only considered LIF-neurons, although all three frameworks are compatible with more biologically realistic neuron models [12, 35, 36]. Moreover, online learning rules for the NEF [37] and Efficient coding [38] have been proposed, but are not considered here. To verify that our claim allows for biological constraints, we created an example by making a rather straight-forward modification of the NEF that yields a biologically plausible weight matrix. Doing this however requires solving Eq 25 explicitly which is computationally expensive, and more sophisticated methods exists for enforcing Dale’s law in the NEF [39].

Elsayed and Cunningham [40] recently argued that neural modes might not be unexpected given long-known properties of tuning and correlation of neurons. Indeed, while the columns of *Z* in Eq 1 correspond to neural modes, the *rows* of *Z* correspond to preferred directions. That is, if the latent variables happen to be analogous to some behaviorally measurable quantities, neuron *j* will exhibit classical cosine-tuning [41] with respect to the angle between the vector given by row *j* of *Z* and the vector of latent variables **x**. This raises the philosophical question of whether we see neural modes because the neurons are tuned, or whether we see neuron tunings because the network has neural modes. In either case, we argue that areas known to exhibit population vector codes are particularly likely to be subject to the kind of architectural framework treated in this work. Notably, one such area is the primary motor cortex [41], which is the area Sadtler et al. [4] recorded from. In conclusion, the approach we have taken here provides a relation between the synaptic weights and not only neural modes but also the general phenomenon of population vector codes.

## Methods

We used three different frameworks for constructing the networks: a Liquid State Machine with FORCE learning, the Neural Engineering Framework and Efficient Coding. The purpose of employing all three frameworks is to demonstrate that they all relate to neural modes in a similar fashion. Because we did not choose comparable parameters for the three types of networks, the differences in their performances (in for instance Fig 2) are not an indicative of their intrinsic ability.

All three frameworks require some tuning of parameters to achieve acceptable performance. The most important one is the time constant *τ*_syn_ of the synaptic kernel. For all synapses (except the fast synapses in Efficient Coding), we used a simple exponential kernel

$$H\left(t\right)=\{\begin{array}{cc} 0 & \text{if}\;t<0\\ e^{-\frac{t}{\tau_{syn}}} & \text{if}\;t\geq 0 \end{array}$$ (28)

A longer time constant greatly improved both the smoothness of the synaptic currents *I*_*j*_ and the memory of the circuit, thus reducing noise and drift of the latent variables.

### FORCE learning

We implemented the network described by Nicola and Clopath [12]. We normalized the units of Eq 2 so that

$$R_{m}=1\;V_{\text{th}}=0\;V_{\text{reset}}=-1\;V_{\text{leak}}=0$$ (29)

Setting the reverse potential at the threshold causes the neurons to be tonically active, removing the need of a driving current to start the network.

Following [12] we set the membrane time constant to *τ*_*m*_ = 10 ms, the synaptic time constant to *τ*_syn_ = 20 ms, and added a *t*_ref_ = 2 ms refractory time after each spike during which the membrane voltage was clamped to *V*_reset_. Each element in the reservoir weight matrix Ω had a 90% probability of being set to 0, and is otherwise drawn from a normal distribution with zero mean and standard deviation

$$\sigma =\frac{G}{0.1*\sqrt{N}}$$ (30)

where *G* = 10 and *N* = 1000. In addition, the reservoir weights were balanced so that the average input weight to each neuron was 0.

The elements of encoder matrix *K* were drawn uniformly from [−100, 100] and the initial decoders Φ(0) where 0. The helper matrix *P* was initialized to

$$P\left(0\right)=5\cdot 10^{-6}I$$ (31)

The decoders and the the helper matrix were subsequently updated every 10th time-step when learning was active, using the following update rules:

Φ(t)=Φ(t-Δt)-e(t)P(t)u(t) (32)

$$P\left(t\right)=P\left(t-\Delta t\right)-\frac{P\left(t-\Delta t\right)u\left(t\right)u^{T}\left(t\right)P\left(t-\Delta t\right)}{1+u^{T}\left(t\right)P\left(t-\Delta t\right)u\left(t\right)}$$ (33)

where **e**(*t*) = **x**(*t*) − **x**_target_(*t*) is the error in the current decoding. The helper matrix $P\left(t\right)\in R^{N\times N}$ is an online estimate of the inverse of the correlation matrix of **u**(*t*) (see [12]).

The network was simulated for a total of 50 seconds for each experiment, with time-step 0.05 ms. During the first 45 seconds, the network was repeatedly exposed to the 2.5 second long target pattern (the steps shown in Fig 2A and a sine and cosine for Fig 4) while learning (Eq 33) was active. During the last 5 seconds learning was not active and the last 2.5 seconds are shown in Fig 2A.

Learning was made more difficult in Fig 2 because of the instantaneous jumps in the target signal. To compensate for this, the network was first trained without the feedback connection. Instead, the target signal was used as input to the network. The feedback was then gradually reintroduced until the network was able independently keep the variables stable after the imposed jumps of latent variables. Note that because the illustration in Fig 2A is using the same latent variable values as was used during training, it is not an unbiased performance test.

### Neural Engineering Framework

We used the NEF implementation in the simulator package Nengo [42] (version 2.6.0). We kept the default parameters of Nengo, but changed the synaptic time constant to *τ*_syn_ = 10 ms (membrane time constant was left at *τ*_*m*_ = 20 ms). Similarly, we used Nengo’s default way of choosing encoders *K* which is to draw each row uniformly from the unit sphere. However, we changed the *max_rates* parameter that controls the scaling of *K* so that the maximal firing rate of each neuron fell between 80 and 120 Hz. From the maximal firing rates the *bias* (*I*_drive_ in Eq 7) and the *gain* were determined automatically by Nengo for each neuron.

The simulation in Nengo is done in two steps. First, an estimated solution of Eq 8 is found and used to connect the network. Second, the network is simulated. To estimate the solution of Eq 8, Nengo draws a number of *evaluation points* from the space of allowed values of **x**_target_. We used Nengo’s default space of allowed values, -1 to 1 for each latent variable, as well Nengo’s default heuristic to determine the number of evaluation points, which gave 2000 points. For each point, Nengo estimates what the expected filtered spike trains **u**(*t*) would be for that point, and then find the least-squares optimal solution to Eq 8 where the average is taken over the evaluation points.

To simulate fixation of variables during the shaded periods in Fig 2B, we connected the *ensemble* in Nengo to a *node* that was in turn connected back to the ensemble. The node was programmed to output the fixed signals during the shaded periods and to simply relay its input otherwise. In the non-shaded periods this is equivalent to recurrently connecting the ensemble to itself. We simulated the network for 2.5 seconds using the default Nengo time-step 1 ms.

### Efficient coding

We implemented the network described by Boerlin et al. [15]. We normalized the membrane voltage so that *V*_leak_ = 0 and *R*_*m*_ = 1. The threshold was set individually for each neuron to

$$V_{\text{th}}^{j}=\frac{\nu \lambda_{syn}+\mu \lambda_{syn}^{2}+∥k_{j}{∥}^{2}}{2}$$ (34)

where *ν* = 10^−3^, *μ* = 10^−6^ and **k**_*j*_ is the *j*th row of *K*. No explicit reset is needed after a spike. Instead, the fast reservoir connections includes inhibitory autapses (from Eqs 10 and 11) which decrease *V*_*m*_ by $\mu \lambda_{syn}^{2}+∥k_{j}{∥}^{2}$ after each spike. We used the same membrane time constant *τ*_*m*_ = 50 ms as [15], but chose a shorter synaptic time constant *τ*_syn_ = 20 ms ⇒ λ_syn_ = 50 Hz.

For Fig 2G, the latent variables actually used in the simulation were 100 times bigger than indicated on the axis. However, since the units of the latent variables are arbitrary we scaled it down in the plot to make comparison easier between the frameworks. To create the figure we simulated the network for 2.5 seconds with time-step 0.1 ms.

### Customized learning rule for NEF

To demonstrate that our result can generalize to a biologically plausible weight matrix we modified Nengo to solve Eq 25 instead of Eq 8 during its building phase. We implemented this modification as a subclass of the *Solver* class in Nengo. Our subclass takes as input a matrix *C* and when called from Nengo, it uses the scipy routine *nnls* to solve the constrained optimization in Eq 25. Similarly, when solving Eq 8, the average in Eq 25 is estimated over a number of evaluation points. In the biologically plausible case, we explicitly increased the number of evaluation points to 40,000.

We did a few more changes to increase the biological plausibility compared to the previous simulations. First, we increased the network size to *N* = 5000 neurons and the number of latent variables to *D* = 4. We also chose a non-linear dynamical system for the latent variables to follow. Namely, we chose *f*(⋅) so that the first two encoded values would be the leading and lagging components of a 2 Hz oscillator and the last two would similarly be the lead and lag of a 4 Hz oscillator. Additionally, we added a non-linear term causing the amplitude to converge to 1. With these terms combined, the latent variables **x**(*t*) evolves as:

$$\begin{array}{c} \begin{array}{cc} \dot{x_{1}} & =\omega x_{2}+\alpha (1-\sqrt{x_{1}^{2}+x_{2}^{2}})x_{1}\\ \dot{x_{2}} & =-\omega x_{1}+\alpha (1-\sqrt{x_{1}^{2}+x_{2}^{2}})x_{2}\\ \dot{x_{3}} & =2\omega x_{4}+\alpha (1-\sqrt{x_{3}^{2}+x_{4}^{2}})x_{3}\\ \dot{x_{4}} & =-2\omega x_{3}+\alpha (1-\sqrt{x_{3}^{2}+x_{4}^{2}})x_{4} \end{array} \end{array}$$ (35)

We used *α* = 0.2 which is sufficient to help stabilize the dynamics to oscillators with frequencies 2 and 4 Hz even in the absence of structured inputs. In the simulations shown in Fig 6 and S2 Fig, we initialized the latent variables *x*_1_…*x*_4_ to 0.

### Simulation tools

Simulation and training of networks designed according to Liquid State Machine and Efficient Coding frameworks were performed using custom scripts written in Julia [43]. Networks designed according to the NEF were implemented using NENGO [42].

### Measuring similarities

In Fig 2C, 2F and 2I, we want to measure the similarity between the subspaces spanned by each pair of matrices (*K*, Φ^*T*^, *Z*_PCA_ and *Z*_PCA_). To this end we measured the cosine of the mean principal angles (as computed by the scipy routine *subspace_angles*, see also [44]). This value ranges from 0 (unrelated, orthogonal space) to 1 (exactly the same space). This measure was also used in S2C Fig.

In S1A, S1B and S1C Fig on the other hand, we sought to demonstrate that the neural modes have indeed been permuted along with the encoders. This change is happening *within* the manifold/subspace, so angles between subspaces are no longer meaningful. Therefore, specifically for S1A, S1B and S1C Fig, we instead calculated the average of the absolutes of the correlations between the columns:

$$sim\left(A,B\right)=\frac{1}{2}(|\text{corr}\left(A_{1:1000,1},B_{1:1000,1}\right)|+|\text{corr}\left(A_{1:1000,2},B_{1:1000,2}\right)|)$$ (36)

Finally, for comparing weight matrices in Figs 3–5, we calculated the correlation over all pairs of old and new weights (i.e. element-wise). We have also reported the Frobenius norm of the difference in S1D and S2D Figs.

## Supporting information

S1 TextIn this text we describe how inside manifold perturbations would affect the connectivity matrix *W* when the perturbation matrix $\tilde{Q}$ is non-orthogonal.This analysis complements the analysis shown in Eqs 13–15 in the main text.(PDF)

S1 Fig Supplementary to Fig 3. (**A**) Pairwise correlations (see Methods) between the four matrices before (subscript “orig” for “original”) and after (subscript “pert” for “perturbed”) an inside-manifold permutation for the FORCE network. (**B**) Same as (A), for the NEF. (**C**) Same as (A), for the Efficient coding framework. (**D**) The same data as in Fig 3C, but with the Frobenius (L2) norm of the difference between the matrices instead of correlation.
(TIF)

S2 Fig Supplementary to Fig 5. (**A**) Spike times of 80 excitatory and 20 inhibitory neurons for the first 2.5 seconds of the simulation. (**B**) The variance explained by the principal components of the binned spike trains. Note that there are four clear dimensions in spite of the fact that the weight matrix has more the four singular values (Fig 5B). (**C**) The cosine of the mean principal angle between the subspace spanned by the columns of each pair of matrices. (**D**) Same data as in Fig 5E, but with Frobenius (L2) norm instead of correlation and with the difference between networks with the same encoders (i.e. no perturbation, red).
(TIF)

S3 Fig Effect of varying the number of dimensions and number of sampled neurons. (**A**) Extension of Fig 3C to higher dimensions. Here we show results for an NEF-network after no perturbation, an inside-manifold permutation and an outside-manifold permutation (other perturbations and networks from Fig 3C are not shown). Error bars indicate standard deviation across five different realizations of the respective perturbation. As the number of latent variables increases, different realizations of the same network start to differ more. However, this difference is not larger for inside-manifold perturbations than unperturbed reinstantiation. Outside-manifold perturbation require big changes (red line) irrespective of the number of latent variables. This is similar to what was shown in in Fig 3C. (**B**) Same data as shown in S2B Fig, but when only using a random subset of the neurons to calculate the principal components. For each number, an independent subset of neurons was selected. Note that subsampling in this fashion does not influence the dimensionality as long as *N* ≫ *D*. In particular, note that dimensionality is an estimation of the rank of the matrix of spikes per bin per neuron (number of bins × nubmer of neurons). Subsampling the columns of this matrix can only decrease the rank, i.e. the dimensionality.
(TIF)
