# Correction: Perturbing low dimensional activity manifolds in spiking neuronal networks

**DOI:** 10.1371/journal.pcbi.1013636

**Published:** 2025-11-03

**Authors:** Emil Wärnberg, Arvind Kumar

An additional affiliation is missing for the second author. Arvind Kumar is also affiliated with the Science for Life Laboratory, Solna, Sweden.

## References

[1] Wärnberg E, Kumar A. Perturbing low dimensional activity manifolds in spiking neuronal networks. PLoS Comput Biol. 2019;15(5):e1007074. doi: 10.1371/journal.pcbi.1007074 31150376 PMC6586365

