# Supplementary material for: Perturbing low dimensional activity manifolds in spiking neuronal networks
Source: PLoS Comput Biol. 2019 May 31;15(5):e1007074. doi: 10.1371/journal.pcbi.1007074 (PMC6586365; doi:10.1371/journal.pcbi.1007074)
Supplement: S1 Text — This analysis complements the analysis shown in Eqs 13–15 in the main text. (PDF) [file pcbi.1007074.s001.pdf]

Perturbing Low Dimensional Activity Manifolds in Spiking Neuronal Networks: Supplementary Information

Emil Wärnberg[1,2], Arvind Kumar[1*]

1 Dept. of Computational Science and Technology, School of Electrical Engineering and Computer Science, KTH Royal Institute of Technology, Stockholm, Sweden
2 Dept. of Neuroscience, Karolinska Institutet, Stockholm, Sweden

Non-orthogonal inside-manifold perturbations

In the derivation of inside-manifold perturbations, we assume that $\tilde{Q}$ is orthogonal. This corresponds to a rigid transformation of the manifold that can include rotations and mirrorings. If we allow for a more general linear transformation that may also include scaling and skewing of the manifold, the orthogonality argument no longer applies and we can no longer expect an inside-manifold perturbation to leave the weights approximately unchanged.

The 2-dimensional case

Consider a general inside-manifold perturbation matrix $Q \in \mathbb{R}^{2\times 2}$ with elements given by

$$Q = \begin{bmatrix} q_{11} & q_{12} \\ q_{21} & q_{22} \end{bmatrix} \tag{S1}$$

From Eq 17 in the manuscript, we expect that an inside-manifold perturbation would give a new weight matrix $\tilde{W} = KQQ^T\Phi$. The crucial step for this operation is the matrix QQ^T, which in the 2-dimensional case becomes

$$QQ^T = \begin{bmatrix} q_{11}^2 + q_{12}^2 & q_{11}q_{21} + q_{12}q_{22} \\ q_{11}q_{21} + q_{12}q_{22} & q_{12}^2 + q_{22}^2 \end{bmatrix} \tag{S2}$$

This gives the perturbed matrix weights

$$\tilde{w}_{ij} = (q_{11}^2 + q_{12}^2)k_{i1}\phi_{1j} + (q_{11}q_{21} + q_{12}q_{22})(\phi_{1j}k_{i2} + \phi_{2j}k_{i1}) + (q_{21}^2 + q_{22}^2)k_{i2}\phi_{2j} \tag{S3}$$

to compare with unperturbed matrix weights

$$w_{ij} = k_{i1}\phi_{1j} + k_{i2}\phi_{2j} \tag{S4}$$

The similarity between $\tilde{w}_{ij}$ and w_{ij} is therefore heavily dependent on the choice of Q.

Elements drawn from a normal distribution

If the elements of Q are drawn from $\mathcal{N}(0, 1)$, the expected $\tilde{w}_{ij}$ from Eq S3 becomes

$$\begin{aligned} \mathbb{E}\left[\tilde{w}_{ij}\right] &= \mathbb{E}\left[q_{11}^2 + q_{12}^2\right] k_{i1}\phi_{1j} + \\ &\quad \mathbb{E}\left[q_{11}q_{21} + q_{12}q_{22}\right](\phi_{1j}k_{i2} + \phi_{2j}k_{i1}) + \\ &\quad \mathbb{E}\left[q_{21}^2 + q_{22}^2\right] k_{i2}\phi_{2j} \\ &= \mathbb{E}\left[\chi^2(2)\right] k_{i1}\phi_{1j} + \\ &\quad 0 \cdot (\phi_{1j}k_{i2} + \phi_{2j}k_{i1}) + \\ &\quad \mathbb{E}\left[\chi^2(2)\right] k_{i2}\phi_{2j} \\ &= 2k_{i1}\phi_{1j} + 2k_{i2}\phi_{2j} \\ &= 2w_{ij} \end{aligned} \tag{S5}$$

The expected weight change after an inside-manifold perturbation with a normally random matrix is thus a factor of 2. This can also be seen directly from the fact that the expected matrix in Eq S2 becomes $2I$. Had the elements of Q instead been drawn from from a normal distribution with $\sigma^2 = 1/2$ the expected weight change after the perturbation would have been 0. Note however, that although for the average perturbation matrix Q chosen this way the perturbed weights will be as similar to the original weights as for an orthogonal perturbation matrix, for most choices of Q the deviation from the original weight matrix will be greater than with an orthogonal Q.

For a perturbation matrix of dimension D, the expected matrix $\mathbb{E}[QQ^T]$ will be D on the diagonals and 0 otherwise, leading to the expected $\mathbb{E}[\tilde{w}_{ij}] = Dw_{ij}$.

All elements equal

Assume that instead of having normally distributed elements, all elements of Q were identical, i.e.

$$Q = \begin{bmatrix} q & q \\ q & q \end{bmatrix} \tag{S6}$$

for some real scalar q, Eq S2 gives

$$QQ^T = \begin{bmatrix} 2q^2 & 2q^2 \\ 2q^2 & 2q^2 \end{bmatrix} \tag{S7}$$

from which it follows that

$$\tilde{w}_{ij} = 2q^2 w_{ij} + 2q^2(\phi_{1j}k_{i2} + \phi_{2j}k_{i1}) \tag{S8}$$

If the elements of K are independently and identically distributed with mean zero, the distribution of $\phi_{1j}k_{i2} + \phi_{2j}k_{i1}$ will be the same as the distribution of w_{ij}. In this case, one can expect that for a manifold perturbation given by Eq S6, about half of the variance of the perturbed weight matrix $\tilde{W}$ can be explained by the original weight matrix W.

Scaling

A consequence of choosing a non-orthogonal Q is that the radius of QQ^T is not necessarily 1. The simplest case of this is setting

$$Q = \begin{bmatrix} s & 0 \\ 0 & s \end{bmatrix} \tag{S9}$$

Naively, applying this transformation would just scale the weight matrix by a factor of s^2. This would not change the neural modes per se, as they are usually defined to have unit norm. Instead, it would be the amplitude of the latent variables that is increased, or, equivalently, the average firing rate of the network.

However, the firing rates of all networks do also depend on other parameters. Arguably, keeping them constant while scaling the synaptic weights might not be realistic, because we expect firing rates to be within some biologically plausible range. In fact, in the NEF implementation Nengo citenengo, the optimization step for finding Φ is set so that the resulting firing rates should be within some target range, rather than scale with K. Thus, scaling K by a factor s would rather correspond to scaling Φ by a factor of $1/s$.

In conclusion, an experiment in which K is scaled would not test the animal's ability to relearn an inside-manifold perturbation, but rather its ability to modulate the global firing rate of the circuit.
