# Supplementary figures and images for: Perturbing low dimensional activity manifolds in spiking neuronal networks
Source: PLoS Comput Biol. 2019 May 31;15(5):e1007074. doi: 10.1371/journal.pcbi.1007074 (PMC6586365; doi:10.1371/journal.pcbi.1007074)

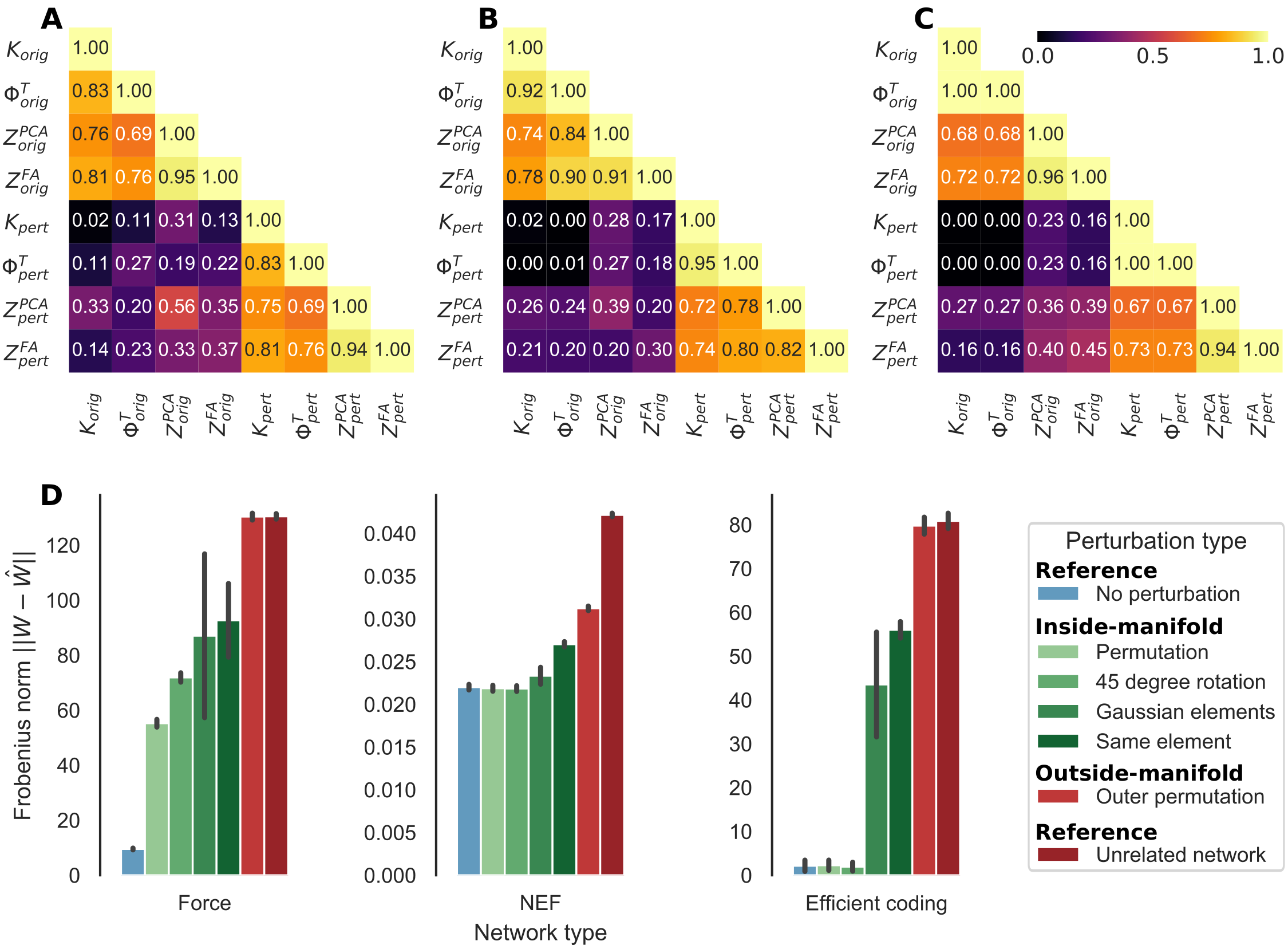

Supplement: S1 Fig — (A) Pairwise correlations (see Methods) between the four matrices before (subscript “orig” for “original”) and after (subscript “pert” for “perturbed”) an inside-manifold permutation for the FORCE network. (B) Same as (A), for the NEF. (C) Same as (A), for the Efficient coding framework. (D) The same data as in Fig 3C, but with the Frobenius (L2) norm of the difference between the matrices instead of correlation. (TIF) [file pcbi.1007074.s002.tif]

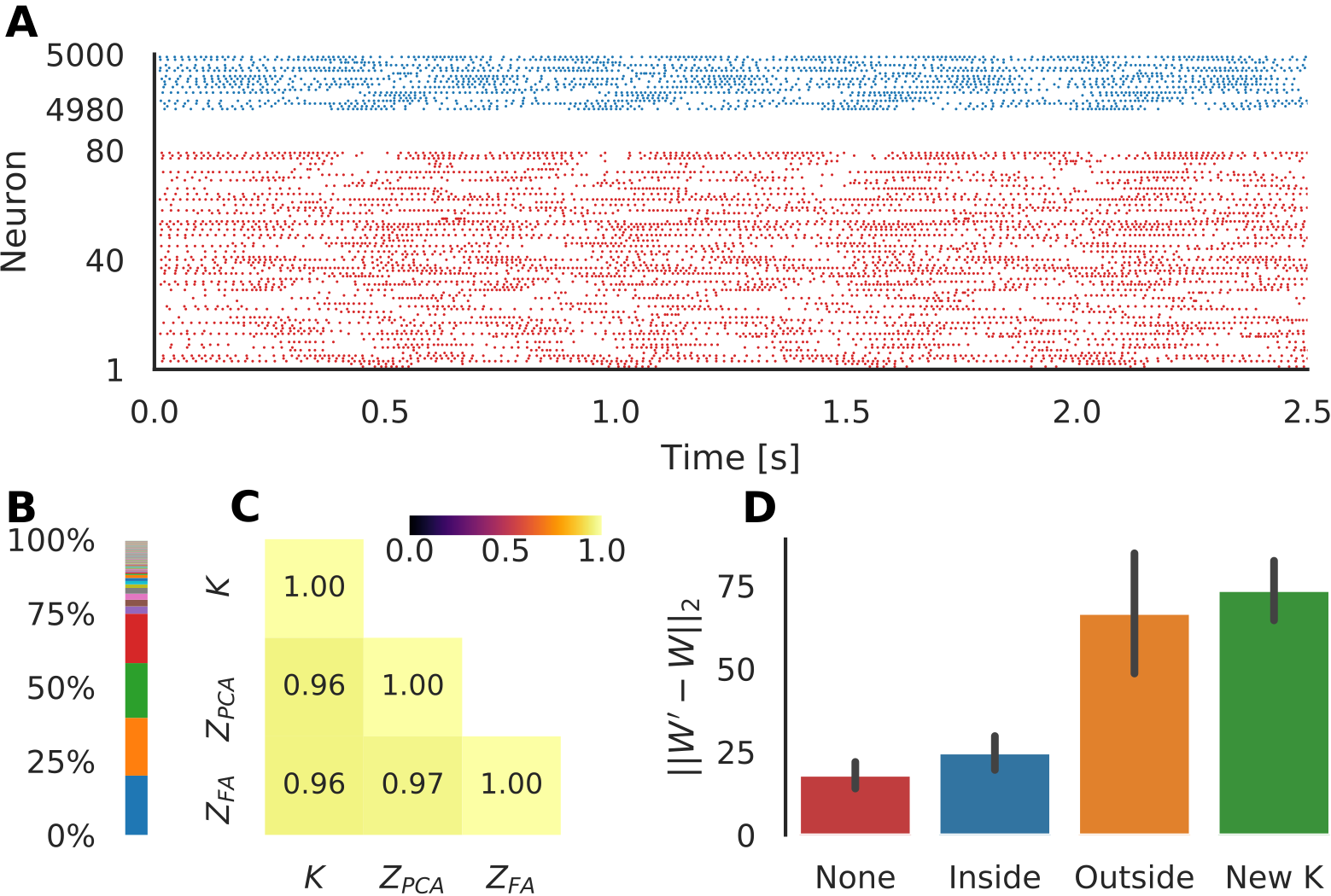

Supplement: S2 Fig — (A) Spike times of 80 excitatory and 20 inhibitory neurons for the first 2.5 seconds of the simulation. (B) The variance explained by the principal components of the binned spike trains. Note that there are four clear dimensions in spite of the fact that the weight matrix has more the four singular values (Fig 5B). (C) The cosine of the mean principal angle between the subspace spanned by the columns of each pair of matrices. (D) Same data as in Fig 5E, but with Frobenius (L2) norm instead of correlation and with the difference between networks with the same encoders (i.e. no perturbation, red). (TIF) [file pcbi.1007074.s003.tif]

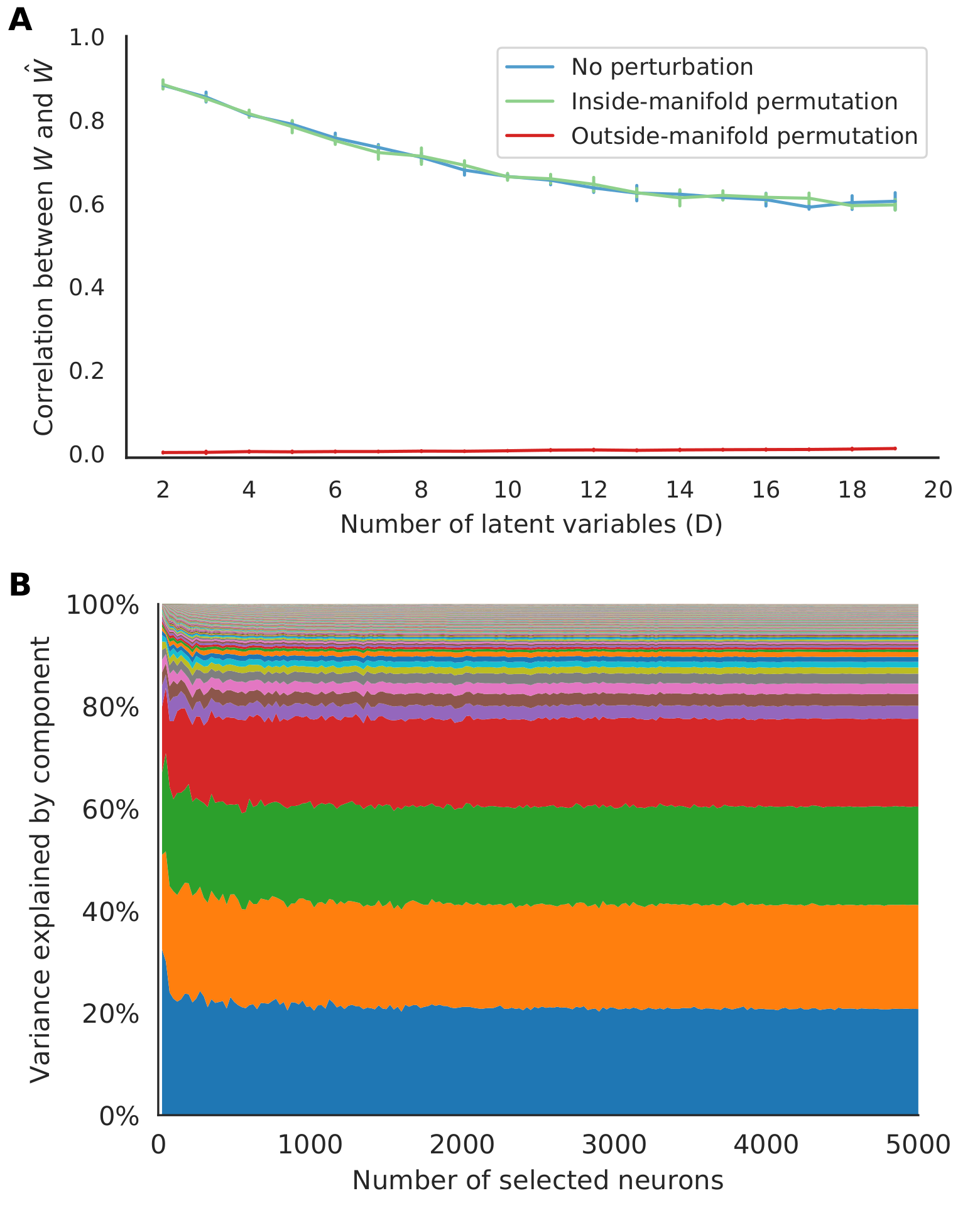

Supplement: S3 Fig — (A) Extension of Fig 3C to higher dimensions. Here we show results for an NEF-network after no perturbation, an inside-manifold permutation and an outside-manifold permutation (other perturbations and networks from Fig 3C are not shown). Error bars indicate standard deviation across five different realizations of the respective perturbation. As the number of latent variables increases, different realizations of the same network start to differ more. However, this difference is not larger for inside-manifold perturbations than unperturbed reinstantiation. Outside-manifold perturbation require big changes (red line) irrespective of the number of latent variables. This is similar to what was shown in in Fig 3C. (B) Same data as shown in S2B Fig, but when only using a random subset of the neurons to calculate the principal components. For each number, an independent subset of neurons was selected. Note that subsampling in this fashion does not influence the dimensionality as long as N ≫ D. In particular, note that dimensionality is an estimation of the rank of the matrix of spikes per bin per neuron (number of bins × nubmer of neurons). Subsampling the columns of this matrix can only decrease the rank, i.e. the dimensionality. (TIF) [file pcbi.1007074.s004.tif]
